# Optimization of variables for cadmium and copper removal using magnetic nanocomposite

**DOI:** 10.1186/s13065-025-01502-5

**Published:** 2025-05-18

**Authors:** Chou-Yi Hsu, Mohammed Ahmed Mustafa, Ghadir Kamil Ghadir, Pooja Bansal, Harpreet Kaur, Amjed Qasim Mohammed, Alzahraa S. Abdulwahid, Ahmed Remthan Hussein, Sahira Qasim Namaha, Ahmed Ali Ami, Usama Kadem Radi, Laith H. Alzubaidi, Ali Kazemi

**Affiliations:** 1 Department of Pharmacy, Chia Nan University of Pharmacy and Science, Tainan City, 71710 Taiwan; 2 Department of Biology, College of Education, University of Samarra, Samarra, 34010 Iraq; 3 Department of Computer Engineering, Institute of Graduate Programs, Al-Turath University, Al Mansour, Baghdad, 10013 Iraq; 4 Department of Biotechnology and Genetics, Jain (Deemed-to-Be) University, Bengaluru, Karnataka 560069 India; 5 Department of Allied Healthcare and Sciences, Vivekananda Global University, Jaipur, Rajasthan 303012 India; 6 School of Basic & Applied Sciences, Shobhit University, Gangoh, Uttar Pradesh 247341 India; 7 Department of Health & Allied Sciences, Arka Jain University, Jamshedpur, Jharkhand 831001 India; 8 Department of Dentistry, Al-Manara College for Medical Sciences, University of Misan, Amarah, Maysan Iraq; 9 Department of Medical Engineering, Al-Hadi University College, Baghdad, 10011 Iraq; 10 Faculty of Science, Mazaya University College, Nasiriyah, Iraq; 11 College of Dentistry, Al-Esraa University, Baghdad, Iraq; 12 Department of Medical Laboratories Technology, Al-Nisour University College, Baghdad, Iraq; 13 College of Pharmacy, National University of Science and Technology, Nasiriyah, Iraq; 14 College of Technical Engineering, The Islamic University of Najaf, Najaf, Iraq; 15 College of Technical Engineering, The Islamic University of Al Diwaniyah, Al Diwaniyah, Iraq; 16 College of Technical Engineering, The Islamic University of Babylon, Babylon, Iraq; 17 School of Public Health, Tehran University of Medical Sciences, Tehran, Iran

**Keywords:** Cadmium, Copper, Optimization, Removal

## Abstract

This study aims to investigate cadmium and copper ultrasound-assisted removal efficiency on a laboratory scale using a cobalt ferrite/activated carbon (COF/AC) composite as an adsorbent. For this purpose, the effect of four independent variables (i.e., composite amount, pH, heavy metal concentrations, and ultrasound radiation time) on the performance of the cadmium and copper removal was investigated. The COF/AC composite was characterized using scanning electron microscopy (SEM), energy-dispersive X-ray (EDX), Brunauer–Emmett–Teller (BET), X-ray diffraction (XRD), and vibrating-sample magnetometer (VSM). The SEM and XRD techniques showed the successful synthesis of the COF/AC composite. The COF/AC composite has a surface area of 659.4 m^2^ g^−1^, an average diameter of 3.6 nm, and a pore volume of 0.482 cm^3^ g^−1^. In this study, R^2^ ˃ 0.99 and Adj-R^2^ ˃ 0.98 for both analytes signify a high agreement between the obtained laboratory data and the model-predicted data. The analysis results for heavy metal removal revealed the following optimal conditions: the composite content of 0.22 g, ultrasound radiation time of 22 min, concentration of 19 mg L^−1^, and pH of 5. Under optimal conditions, the maximum removal efficiency reached 93.46% and 97.45% for cadmium and copper, respectively. The COF/AC composite reuse results showed no significant decrease in removal efficiency up to 4 times of use during the adsorption and desorption process. Analysis of real samples showed that the removal rates of cadmium and copper were 89.62% and 96.37%, respectively.

**Supplementary Information:**

The online version contains supplementary material available at 10.1186/s13065-025-01502-5.

## Introduction

A wide range of chemical pollution in air, water, soil, and sediments threatens our environment. Industrial wastewater produces a huge volume of organic and inorganic pollutants [1]. In recent years, metal pollution in aquatic environments has gained significant attention due to its toxicity, persistence, and environmental stability [2]. Wastewater from various industries (e.g., textile, printing, food, and cosmetics) is a source of excessive heavy metals entering the environment [3].

Cadmium is a heavy and toxic metal with a half-life of approximately 30 years in bones and carcinogenic properties [4]. Cadmium accumulation in the human body causes hemolysis, nausea, increased salivation, muscle contractions, kidney damage, chronic lung problems, and skeletal deformities [5]. The World Health Organization (WHO) has established a maximum permissible cadmium concentration of 0.002 mg L^−1^ in drinking water [6].

Copper is one of the most abundant metal pollutants widely used across various industries, including metal plating and polishing, paper milling, wood pulp production, and paint and fertilizer manufacturing [7, 8]. Accumulation of copper in the human body can lead to stomach ulcers, skin disorders, and liver and brain damage. The WHO has established a maximum acceptable copper concentration of 0.05 mg L^−1^ in drinking water and a permissible discharge limit of 1 mg L^−1^ to surface water [9].

Several techniques (e.g., chemical coagulation, adsorption using various adsorbents, ion exchange, reverse osmosis, nanofiltration, electrocoagulation, and biological processes) have been applied to remove pollutants from aqueous environments [10–15]. Adsorption offers several advantages, including lower surface area requirements, reduced sensitivity to daily fluctuations, resistance to toxic chemicals, high pollutant removal efficiency, and greater flexibility in design and operation compared to other wastewater treatment methods [16, 17].

Ferrite nanoparticles (Fe_2_O_4_ NPs) are widely used due to their large surface area and high adsorption capacity [18]. Cobalt ferrite (CoFe_2_O_4_) is a hard magnetic material with an inverse spinel structure [19]. These nanoparticles have attracted significant attention because of their unique properties, including magnetic anisotropy, high solubility, large surface area, high chemical stability, and greater pore size and volume than other ferrites [20]. Additionally, CoFe_2_O_4_ is a valuable adsorbent for purifying samples containing various pollutants. CoFe_2_O_4_ NPs exhibit strong magnetic properties, allowing them to be easily immobilized on any substrate to expedite the separation process. Furthermore, they do not produce secondary pollution and eliminate the need for filtration or centrifugation [21].

Activated carbon is a group of carbon-based materials with a high internal surface area. It is considered a unique material due to its extensive internal area, hollow and porous structure, high adsorption capacity, surface reactivation ability, and low cost compared to inorganic adsorbents [22, 23]. Because of its porous structure and large surface area, activated carbon has recently been used as a substrate for the uniform distribution of iron oxide nanoparticles (NPs). Since most metal oxide NPs tend to accumulate and aggregate, their active surface area can be reduced, limiting their effectiveness in catalytic reactions [24]. Embedding these NPs onto a porous substrate, like activated carbon, is an effective solution to this issue, enabling a uniform distribution of metal oxide NPs and subsequently enhancing their catalytic activity [25].

For instance, Modabberaslu et al. [26] synthesized CoFe_2_O_4_ NPs for azithromycin removal. They examined the effects of nanoparticle content, contact time, azithromycin concentration, and pH on the removal efficiency. Results indicated that the maximum azithromycin removal (> 89%) was achieved with 60 mg of NPs, a pH of 6.67, a contact time of 90 min, and an azithromycin concentration of 20 mg L^−1^ [26]. Similarly, Simonescu et al. [27] utilized a CoFe_2_O_4_-chitosan composite to remove Congo red and methyl orange, reporting maximum adsorption capacities of 66.18 mg g^−1^ for methyl orange and 15.60 mg g^−1^ for Congo red. They identified solution pH, adsorbent content, and contact time as key parameters influencing the adsorption process [27].

Adsorption efficiency is affected by various factors, including pH, contact time, adsorbent content, and analyte concentration. Simultaneous optimization of these factors can enhance adsorption efficiency significantly [28]. Conventional optimization methods in multifactor analysis are time-consuming, require large amounts of chemicals, and often lack accuracy due to high error margins [29]. Furthermore, these methods do not allow for evaluating the interaction effects between variables. Chemometric approaches, such as the response surface method (RSM), can be employed to address these limitations and enable the examination of interaction effects among variables [30].

RSM is a powerful statistical and mathematical technique used for modeling and optimizing complex processes. Unlike conventional experimental designs, RSM evaluates multiple variables’ collective influence and interactions through a structured approach, leading to more efficient identification of optimal conditions [31]. Using this technique minimizes the number of required experiments and enhances the accuracy and robustness of the resulting models, making it especially valuable in process optimization and industrial applications [32].

Therefore, the present study was designed to evaluate the efficiency of COF/AC as an adsorbent for the ultrasound-assisted removal of cadmium and copper from aqueous solutions. Key operational parameters, such as pH, ultrasound exposure time, composite content, and heavy metal concentration, were optimized using RSM. In addition, the potential for adsorbent recycling and reuse and its performance in real water samples were investigated.

## Experimental

### Materials and equipment

All chemicals used throughout the experimental procedures were of analytical grade and employed without any further purification. Reagents including ammonia, copper (II) nitrate trihydrate, sodium hydroxide, cobalt (II) nitrate hexahydrate, cadmium (II) nitrate tetrahydrate, iron (III) nitrate nonahydrate, hydrochloric acid, and ethanol were purchased from Sigma-Aldrich (Germany). The AC was purchased from Fluka (France). A stock solution of heavy metal with a concentration of 1000 mg L^−1^ was prepared in deionized water. The pH of the samples was measured with a Metrohm-914 (Netherlands) pH meter. Also, the concentrations of remaining analytes in the samples were quantified using an Agilent 2100 (Germany) atomic absorption spectrometer. An ultrasonic device (PS-20 A, China) was used to create a mixture between the adsorbent and heavy metal molecules. The surface morphology of the COF/AC composite was characterized through SEM (Philips XL-30, Netherlands). The XRD pattern and the presence of CoFe_2_O_4_ in the activated carbon structure were determined via the XRD analysis (Philips PW 1710, Netherlands). The magnetic properties of the synthesized composite were analyzed by a VSM device (Lake Share Company, 7400, USA). The BET (Quantachrome NOVA-3000, USA) was applied to determine the specific surface area, volume, and size of the COF/AC surface pores. The weight and atomic ratio of the elements in the composite were measured through the EDX analysis.

### Synthesis COF/AC composite

The COF/AC composite was synthesized using the chemical co-precipitation method. First, a solution containing iron and cobalt nitrates in a molar ratio of 2:1 was prepared and stirred under nitrogen gas at 80 ℃ for 30 min. Then, 2.5 g of activated carbon was added to the solution and stirred for 1 h. In the next step, a 25% ammonium solution was added dropwise to the solution to reach a pH of 10, after which the solution was stirred by a stirrer at 50 rpm for 1 h. Finally, the obtained powder was separated from the solution by an external magnet and washed repeatedly with distilled water and then ethanol. Finally, the synthesized adsorbent was oven-dried at 90 ℃ for 4 h. The characteristics of the COF/AC composite were analyzed using SEM, BET, XRD, VSM, and EDX techniques.

### Removal experiments

This study was conducted on synthesized samples on a laboratory scale. The cadmium and copper removal process by the COF/AC composite was analyzed by investigating the effects of composite content (0.01–0.03 g), pH (2–6), ultrasound radiation time (5–25 min), and heavy metal concentrations (10–50 mg L^−1^) on the removal efficiency. The test steps for the studied variables were set according to 30 runs designed by RSM. The pH values ​​were adjusted by adding NaOH (0.1 M) or HCl (0.1 M) solutions. At each step after the removal process, the solid phase (composite) was separated from the solution using an external magnetic field, and the remaining concentration in the solution was measured using an atomic absorption spectrometer. Based on the obtained results, the removal efficiency by the synthesized composite was calculated using Eq. (1).

1 $$\%Removal=\frac{{C}_{0}-{C}_{e}}{{C}_{0}}\times 100$$

where R is the removal efficiency (%), *C*_*0*_ is the initial concentration (mg L^−1^), and *C*_*e*_ is the concentration (mg L^−1^) at equilibrium time.

### Central composite design (CCD)

The dependence of cadmium and copper removal efficiency on concentration, solution pH, ultrasound radiation time, and composite content has been established in previous reports [33, 34]. In this study, four independent variables, including concentration (A) ranging from 10 to 50 mg L^−1^, pH (B) from 2 to 6, composite content (C) from 0.01 to 0.03 g, and contact time (D) from 10 to 25 min were selected to achieve optimal removal conditions. The actual values of the process variables and their ranges were determined based on preliminary tests (Table 1). The combined effect of these variables on cadmium and copper removal efficiency across five levels was investigated using a statistical design in Stat-Ease Design-Expert v12 software and RSM based on the CCD. For statistical calculations, *Z* was coded as shown in Eq. 2.

2 $$\text{Z}=\frac{{X}_{i}-{X}_{0}}{\Delta X}$$

where *Z* is the coded value of the variable, *X*_*i*_ is the actual value of the variable, *X*_*0*_ is the actual value of *X*_*i*_ at the center point, and *ΔX* is the step change value [35]. Removal efficiency (R%) was considered the dependent variable (response). A functional relationship between the independent variable and the response was modeled using a quadratic polynomial equation (Eq. 3) to explain the variables’ effects in a quadratic and reciprocal linear manner, as shown in Table 1.

3 $${\text{Y}=\beta }_{0}+\sum_{i=1}^{k}{\beta }_{i}{X}_{i}+ \sum_{i=1}^{k}{\beta }_{ii}{X}_{i}^{2}+ \sum_{i\le j}^{k}\sum_{j}^{k}\beta ij{X}_{i}{X}_{j}+\varepsilon$$

where *Y* is the predicted response (output), *β*_*0*_ is the constant regression coefficient, *β*_*i*_ represents the linear effect, *β*_*ij*_ the interaction effect, *β*_*ii*_ the squared effect, and *ε* is the observed error [36].

**Table 1:** The CCD matrix

Variables	Unit	Symbols	Level of variables					
			− α	− 1	0	+ 1		+ α
Concentration	Mg L^−1^	A	10	20	30		40	50
pH of solution	–	B	2	3	4		5	6
Composite amount	g	C	0.10	0.15	0.20		0.25	0.30
Ultrasound radiation time	min	D	5	10	15		20	25

Regression coefficients were used for statistical calculations and plotting. In the design of experiments, the CCD factorial design model includes *2*^*n*^ factorial points, *2n* axial points, and *n*_*c*_ central points, which is widely used to express variable-response relationships. As shown in Table 2, the tests included 16 factorial points, 8 axial points, and 6 central points, totaling 30 runs, which were generated as an initial design using Stat-Ease Design-Expert software, as per Eq. 4.

4 $$\text{N}={2}^{n}+2n+{n}_{c}$$

where *N* is the total number of required tests and *n*_*c*_ is the number of factors [37].

**Table 2:** The results of CCD matrix

Variables					%R-Cadmium		%R-Copper	
Run	A (mg L^−1^)	B	C (g)	D (min)	Experimental	Predicted	Experimental	Predicted
1	0	0	0	0	72.69	74.19	77.31	77.33
2	0	0	2	0	74.7	73.21	69.64	67.88
3	0	0	0	0	73.5	74.19	73.88	77.33
4	− 1	− 1	− 1	− 1	45.81	47.00	55.65	56.46
5	− 1	− 1	− 1	1	62.78	63.47	73.04	72.86
6	1	− 1	− 1	− 1	31.77	31.88	32.64	32.08
7	1	1	1	1	67.74	66.94	62.17	62.84
8	0	0	0	− 2	46.28	44.24	54.85	54.38
9	0	− 2	0	0	41.06	39.40	44.36	44.01
10	1	1	− 1	1	53.67	54.39	53.04	53.23
11	− 1	1	1	1	91.32	92.85	89.85	91.30
12	0	0	0	0	75.68	74.19	79.16	77.33
13	− 1	1	1	− 1	72.25	72.42	77.09	77.68
14	1	− 1	1	1	55.48	56.93	49.6	50.98
15	0	0	0	0	75.53	74.19	74.71	77.33
16	0	0	0	2	77.74	77.74	82.46	80.56
17	0	0	0	0	72.93	74.19	78.17	77.33
18	0	0	− 2	0	47.68	47.13	46.23	45.62
19	− 1	− 1	1	− 1	59.6	60.53	68.42	69.12
20	− 1	1	− 1	− 1	58.11	58.31	62.26	61.77
21	− 1	− 1	1	1	84.16	83.45	81.8	81.96
22	1	1	1	− 1	48.96	49.91	51.98	53.05
23	− 1	1	− 1	1	73.37	72.29	77.33	78.94
24	0	2	0	0	61.1	60.72	63.19	61.17
25	2	0	0	0	44	42.41	40.18	38.95
26	0	0	0	0	74.81	74.19	80.74	77.33
27	1	− 1	1	− 1	35.93	37.40	42.1	41.97
28	1	1	− 1	− 1	42.7	43.80	38.56	39.89
29	1	− 1	− 1	1	44.74	44.96	43.75	44.64
30	− 2	0	0	0	83.88	83.43	92.94	91.80

## Results and discussions

### Characterization studies

Fig. S1a illustrates the morphology and surface characteristics of the COF/AC composite by SEM. The SEM images of the synthesized nanoparticles reveal relatively uniform morphology and the presence of surface pores. Fig. S1b displays the elemental analysis results of the synthesized catalyst using EDX. The figure also shows the peaks of all the elements in the COF/AC composite structure, consisting of iron, cobalt, oxygen, and carbon. The physical characteristics (i.e., specific surface area, volume, and pore size distribution) of the COF/AC composite were determined using the BET analysis and BJH theories based on nitrogen gas adsorption and desorption isotherms. The average pore size obtained through BET and BJH analyses is 3.2 and 1.3 nm, respectively. Also, the COF/AC composite’s specific surface area was 659.4 m^2^ g^−1^. The adsorbent’s average diameter and total pore volume were equal to 3.6 nm and 0.482 cm^3^ g^−1^, respectively. Fig. S1c depicts nitrogen gas adsorption and desorption isotherms for the synthesized COF/AC composite. Fig. S1 d illustrates the pore size distribution and the pore volume. The crystalline phase of the synthesized COF/AC composite was determined with the XRD analysis using a Cu beam in the angular range of 2θ = 10–70° at 25 ℃. In the XRD pattern of the COF/AC composite (Fig. S1e), the peak at about 2θ = 25° indicates the presence of AC. The peaks appeared in 2θ = 18.9°, 30.2°, 36.1°, 43.2°, 52.3°, 57.7°, and 63.8° correspond to the (111), (220), (311), (222), (400), (422), (511), and (440) indices, respectively. The peaks in the obtained XRD pattern correspond well with standard JCPDS Card no. 22–1086 and show the presence of the cubic spinel phase structure of CoFe_2_O_4_. After drying the synthesized COF/AC composite at room temperature (25 ℃), its magnetic properties were investigated using the VSM analysis (Fig. S1f). The VSM analysis was performed for the sample in the magnetic field range of ± 10 KOe and a saturation magnetization (Ms) range of ± 25 emu g^−1^. The results showed that the Ms was 17 emu g^−1^ for the synthesized COF/AC composite. Therefore, the COF/AC composite can be separated from the solution using an external magnet (magnetic field).

### Determination of point of zero charge (PZC) of adsorbent

**Fig. 1:**
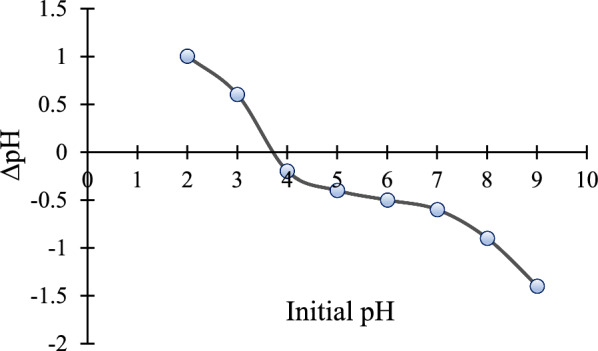
The pH_pzc_ of COF/AC composite

The pH_pzc_ is the point at which the adsorbent surface has a neutral charge. In this research, pH_pzc_ was determined using the solid addition method. To this end, 10 mL of a 0.01 M sodium chloride solution was added to separate vessels, and solutions were prepared at eight initial pH values (ranging from 2 to 9). The pH of these solutions was adjusted using 0.01 M HCl and 0.01 M NaOH. Then, 0.02 g of the adsorbent was added to each solution, and the samples were stirred on a shaker at 150 rpm. After 24 h, the adsorbents were separated, and the pH of each solution was measured again. A graph was plotted based on ΔpH (i.e., the difference between final and initial pH) against the initial pH. The intersection points of ΔpH and initial pH were identified as pH_pzc_. The test results (Fig. 1) indicate a pH_pzc_ value of 3.8 for the COF/AC composite.

### Analysis of variance (ANOVA)

The individual and interaction effect of each variable on the cadmium and copper removal process and the significance of each variable were obtained through the ANOVA. The relationship between the response (removal efficiency) and the variables was obtained using a quadratic equation and multivariate regression analysis of the experimental data based on Eqs. (5) and (6) in coded form.

5 $$\text{\%R}-\text{Cadmium }= +74.19 -10.25\text{A }+5.33\text{B }+6.52\text{C }+8.37\text{D }+0.15\text{A}*\text{B }-2.01\text{A}*\text{C }-0.84\text{A}*\text{D }0.14\text{B}*\text{C }-0.62\text{B}*\text{D }+1.61\text{C}*\text{D }-{2.81\text{A}}^{2} -{6.03\text{B}}^{2} -{3.50\text{C}}^{2} -{3.30\text{D}}^{2}$$

6 $$\text{\%R}-\text{Copper }= +77.32 -13.21\text{A }+4.28\text{B }+5.56\text{C }+6.54\text{D }+0.62\text{A}*\text{B }-0.68\text{A}*\text{C }-0.95\text{A}*\text{D }+0.81\text{B}*\text{C }+0.19\text{B}*\text{D }-0.88\text{C}*\text{D }-{2.98\text{A}}^{2} -{6.18\text{B}}^{2} -{5.14\text{C}}^{2} -{2.46\text{D}}^{2}$$

where *A*, *B*, *C*, and *D* were the initial concentration, pH, composite content, and ultrasound radiation time, respectively.

The importance of each parameter and its interaction effect using the square model in the ANOVA is shown in Table 3. The correlation of each variable with the percentages of cadmium and copper removal was determined by the F-test and p-value. The F-test indicates the effect or lack of effect of the tested parameters at the intended confidence level. The F-value is calculated as the ratio of the mean sum of squares of data to the mean sum of squares of errors. The p-value shows the probability of error in accepting the validity of the observed results, meaning that the probability of the result occurring by chance is not high. For example, a p-value of 0.05 means that 5% of the results may be due to random chance. Therefore, a high F-value and a p-value less than 0.05 indicate the significance of the regression model at the 95% confidence level. The results in Table 3 indicate that the regression models, with p-values less than 0.0001 and high F-values (223.62 and 140.83 for cadmium and copper, respectively), were statistically significant, demonstrating that the models are suitable for spatial design. Moreover, the coefficient of determination (R^2^) values for cadmium (0.9952) and copper (0.9924) indicates that over 99% of the experimental data aligns with the model’s predictions. Additionally, the adjusted R^2^ (Adj-R^2^) values obtained for cadmium (0.9908) and copper (0.9854) are close to the R^2^ values, which further supports the model’s statistical robustness. The p-values for lack of fit for cadmium (0.3314) and copper (0.9009) indicate a significant lack of fit, suggesting an interval between the predicted and actual values, which could point to unaccounted-for systematic variations in the model. Adequate precision represents the signal-to-noise ratio, indicating the extent of variation in results across multiple tests. For this modeling approach, an Adequate precision value above 4 is considered satisfactory. In this study, Adequate precision values were 56.04 and 41.65 for cadmium and copper, respectively.

**Table 3:** The ANOVA of removal of heavy metals

Source	DF	Cadmium				Copper			
		Sum of squares	Mean square	F-value	P-value	Sum of squares	Mean square	F-value	P-value
Model	14	7412.14	529.44	223.62	< 0.0001	8105.48	578.96	140.83	< 0.0001
A	1	2524.99	2524.99	1066.47	< 0.0001	4190.21	4190.21	1019.26	< 0.0001
B	1	681.92	681.92	288.02	< 0.0001	441.53	441.53	107.40	< 0.0001
C	1	1020.90	1020.90	431.19	< 0.0001	743.26	743.26	180.80	< 0.0001
D	1	1684.21	1684.21	711.35	< 0.0001	1028.35	1028.35	250.14	< 0.0001
A*B	1	0.3752	0.3752	0.1585	0.6962	6.30	6.30	1.53	0.2348
A*C	1	64.12	64.12	27.08	0.0001	7.59	7.59	1.85	0.1943
A*D	1	11.54	11.54	4.88	0.0432	14.67	14.67	3.57	0.0784
B*C	1	0.3452	0.3452	0.1458	0.7080	10.66	10.66	2.59	0.1282
B*D	1	6.21	6.21	2.62	0.1261	0.6084	0.6084	0.1480	0.7059
C*D	1	41.57	41.57	17.56	0.0008	12.64	12.64	3.07	0.1000
A^2^	1	217.75	217.75	91.97	< 0.0001	244.91	244.91	59.57	< 0.0001
B^2^	1	998.19	998.19	421.60	< 0.0001	1049.05	1049.05	255.18	< 0.0001
C^2^	1	336.98	336.98	142.33	< 0.0001	725.89	725.89	176.57	< 0.0001
D^2^	1	298.72	298.72	126.17	< 0.0001	166.58	166.58	40.52	< 0.0001
Residual	15	35.51	2.37			61.67	4.11		
Lack of Fit	10	26.80	2.68	1.54	0.3314	27.22	2.72	0.3950	0.9009
Pure Error	5	8.71	1.74			34.45	6.89		
Cor Total	29	7447.65				8167.15			

Model summary statistics							
Cadmium				Copper			
Adeq Precision	R^2^	Adj-R^2^	Pred-R^2^	Adeq Precision	R^2^	Adj-R^2^	Pred-R^2^
56.04	0.9952	0.9908	0.9776	41.65	0.9924	0.9854	0.9747

It is necessary to validate and verify the model to confirm that it approximates the actual system well. Graphical and numerical methods can be employed for this purpose; the graphical approach is used here to examine model residuals. Residuals are defined as the difference between observed and fitted values. A key assumption in regression is the random and normal distribution of residuals. Figs. S2a and S2b show plots of residuals against predicted values. From these figures, the residuals appear to follow a random distribution without any clear pattern. In the normal probability plots of the residuals (Figs. S2c and S2 d), the alignment of points along the straight line and the graph’s linearity suggests a normal distribution of residuals, with the average difference between residuals and actual values close to zero. The correlation between model-predicted and observed values (Figs. S2e and S2f) shows that most predicted values closely match actual values, with only a few exceptions.

### Response surface plots

**Fig. 2:**
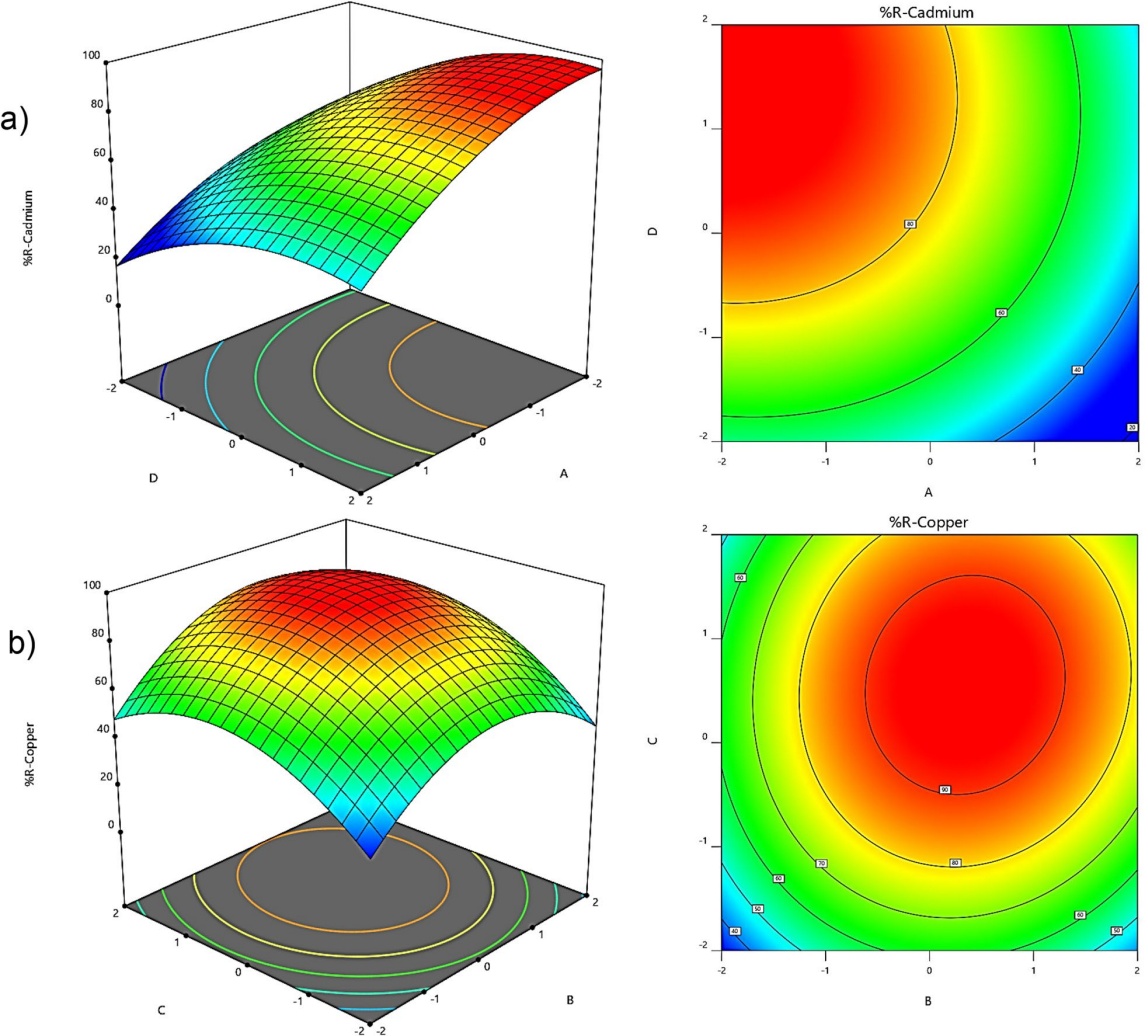
The 3D and 2D graphs of removal of (**a**) cadmium and (**b**) copper

Examining the effects between different factors distinguishes the RSM method from other design models. This feature is well represented by 2D and 3D graphs in Stat-Ease Design-Expert software. These graphs are indeed used to show the relationship between two independent variables and a dependent variable. The values ​​of the independent variables are displayed along the X and Y axes. Contour lines represent dependent variable (Z) values in 2D graphs. In other words, these lines represent different values ​​of X and Y variables that produce identical Z values. In the 3D graphs, the value of the Z variable is displayed by a flat surface along the Z axis instead of the contour lines. Figure 2a and b illustrate the 2D and 3D graphs.

As shown in Fig. 2a, the initial cadmium concentration significantly influences the removal efficiency. The removed cadmium percentages within 30 min for the initial cadmium concentrations of 10 and 50 mg L^−1^ were 85% and 38%, respectively. Fast adsorption in the initial stage arises from a high driving force created by the availability of vast adsorbent surfaces and low cadmium ions. The efficiency reduction with increasing the initial cadmium concentration is attributed to the constant number of adsorption sites on the adsorbent surface in the solution. Thus, fewer sites were available for occupation with the elevated cadmium concentration, thereby lowering efficiency. These results are consistent with those of some previous studies. Sayago et al. [38] presented evidence that heavy metal adsorption on an adsorbent from the aquatic plant *Eichhornia crassipes* reached equilibrium quickly, which is close to our findings [38].

The effect of changes in ultrasound radiation time on the cadmium removal efficiency was studied in the range of 5–25 min (Fig. 2a). According to this figure, more than 91.13% of cadmium was removed in the first 20 min after adding the adsorbent. Also, about a 4% increase was observed in the removal process from 20 to 30 min. Therefore, 20 min was chosen as the optimal time for the removal process. Consequently, the removal efficiency was improved by increasing the ultrasound radiation time because of the increased contact between the adsorbent and heavy metal ions. Consistent with our results, Yang et al. [39] reported that the equilibrium time for removing enrofloxacin and Rhodamine B using graphene oxide was less than 23 min [39].

The pH value of a solution influences the removal of heavy metals using an adsorbent. Therefore, copper adsorption on the COF/AC composite was investigated at different pH values. Figure 2b depicts the change in the copper removal percentage influenced by the solution pH. Copper removal increased significantly with increasing the solution pH and reached the maximum value at pH of 5, which was chosen as the optimal pH for the next experimental stages. The reason for this issue can be explained based on the pH_pzc_ of the adsorbent. If pH > pH_pzc_, the adsorbent surface is negative, and the adsorbent surface is positive if pH < pH_pzc_. In the present study, a pH_pzc_ of 3.8 was obtained for the adsorbent, and the optimum pH (5.0) was higher than the pH_pzc_. Therefore, the adsorbent surface was negative at pH > 3.8, resulting in the increased removal percentage with the increase in pH due to the electrostatic attraction between Cu ions and the adsorbent surface. The maximum removal efficiency was recorded at pH 5.0. At pH values > 6, on the other hand, cadmium and copper are in their hydroxide form and precipitate in the solution. Therefore, pHs > 6 were not used to study the effect of pH on heavy metal removal. Moreover, consistent with the observations of this work, Kandah [40] showed that the adsorption process improved with increasing pH up to an optimal value of 5 [40].

According to the effect of the COF/AC composite content on copper removal efficiency (Fig. 2b), the removal efficiency increased from 40.17% to 90.89% by increasing the COF/AC composite from 0.10 g to 0.22 g. The removal efficiency is almost constant for adsorbent contents ​​more than 0.22 g. The increased copper removal up to the adsorbent value of 0.22 g is caused by the increase in the number of available sites for copper adsorption. No increase in copper removal for adsorbent contents of ​​ > 0.22 g can be explained by the overlap of the adsorption sites on the adsorbent surface, thereby reducing adsorption sites and lowering the adsorption efficiency and rate. In a recent study, Shojaei et al. [41] employed nanozeolite-X to remove methyl orange, reactive blue 15, and reactive red 239. The results revealed a significant increase in removal efficiency with increasing adsorbent amount, followed by stabilization at equilibrium [41].

### Optimization of variables

This study used the desirability function as the numerical optimization method to determine the optimal conditions for the maximum removal of cadmium and copper. Optimizing the combination of variables was essential to achieve the highest removal efficiency. According to the optimal conditions provided by the CCD method and the numerical optimization results, the maximum removal of cadmium and copper is presented in Table S1. Three tests were run under optimal conditions to confirm the model prediction results. Based on the average test results, a good agreement was obtained between the cadmium and copper removal rates predicted by the model and those obtained from the experiments.

### Interference studies

The presence of other adsorbable ions in the solution can compete with target analytes for active adsorption sites on the adsorbent. As a result, these ions may hinder the effective adsorption of the analytes by the adsorbent. To evaluate this, the efficiency of the COF/AC composite for cadmium and copper removal under optimal conditions was tested by conducting experiments in the presence of various ions. The results, shown in Table 4, indicate no significant changes in the COF/AC composite’s ability to remove cadmium and copper. Thus, the presence of different cations and anions in the solution does not substantially affect the COF/AC composite’s removal capability, nor can they effectively compete with cadmium and copper ions.

**Table 4:** Effect of interfering ions on removal of heavy metals

Interference	Tolerance ratio	Cadmium	Copper
${\text{K}}^{+}$	1000	96.34 ± 2.5	97.61 ± 2.2
${\text{F}}^{-}$	1000	96.30 ± 1.9	96.78 ± 1.9
${\text{NO}}_{3}^{-}$	700	97.28 ± 2.3	95.79 ± 1.8
${\text{Fe}}^{2+}$	500	97.12 ± 1.9	96.62 ± 1.7
${\text{Al}}^{3+}$	250	95.94 ± 1.6	97.79 ± 2.0
${\text{Pb}}^{2+}$	100	97.20 ± 2.3	96.68 ± 1.5

### Recovery of adsorbent

**Fig. 3:**
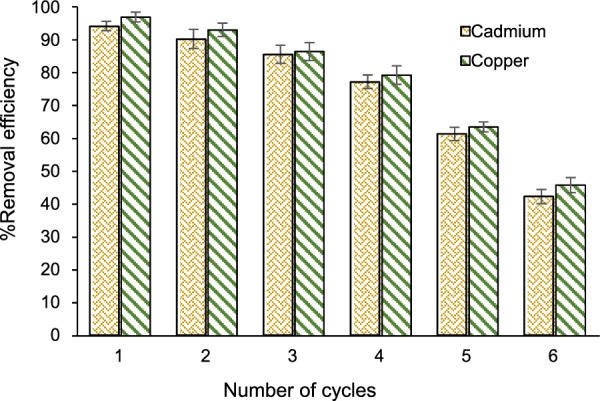
Reusability of the synthesized COF/AC composite

The reuse of the adsorbent is an important factor to consider when synthesizing an adsorbent because it is of paramount importance both in terms of synthesis and economics. To evaluate this, experiments were conducted under optimal conditions (ultrasound radiation time of 20 min, pH of 5, composite content of 0.22 g, and concentration of 19 mg L⁻^1^). After the completion of each process, the COF/AC composite was separated from the solution using a magnet and washed with ethanol. Then, the adsorbent was dried in an oven at 80 ℃ for 3 h to prepare it for reuse in subsequent tests. According to Fig. 3, reusing the COF/AC composite up to 4 times did not significantly reduce its removal efficiency. However, a reduction occurred in the removal efficiency after the fourth reuse, likely because some of the active phases of the COF/AC composite were lost from the substrate surface during washing. Moreover, reactive substances might have deposited on the composite surface, leading to its deactivation.

### Real samples analysis

The COF/AC composite performance in cadmium and copper removal from different water samples was evaluated using several synthetic samples prepared from drinking, surface, and wastewater. To prepare the synthetic samples, specific amounts of heavy metal ions were added to a fixed volume of water samples, and tests were performed as described in Sect. Conclusion. The results of each triplicate test are presented in Table 5. The cadmium and copper removal results from the synthesized samples revealed the high efficiency of the COF/AC composite in removing these metals from real samples. The results demonstrated the ability of the synthesized adsorbent to remove pollutants from environmental water samples.

**Table 5:** Removal of heavy metals in environmental water samples

Samples	Cadmium	Copper
Drinking water	92.81 ± 2.2	96.37 ± 1.8
Surface water	94.47 ± 2.5	96.12 ± 2.1
Wastewater	89.62 ± 2.2	91.69 ± 2.7

### Comparison with other methods

Table 6 compares the performance of the COF/AC composite with other adsorbents for cadmium and copper removal, suggesting that the COF/AC composite is competitive with those reported in the literature. The ultrasonic process significantly improved the process efficiency, and the process time in our study was shorter than that reported in other recent studies. The COF/AC composite was used in lower quantities than the other adsorbents for cadmium and copper removal. Moreover, using the experimental design method reduced the number of tests, saved money and time and increased cost-effectiveness compared to other existing methods. Accordingly, the COF/AC composite showed satisfactory performance in cadmium and copper removal from environmental water samples.

**Table 6:** Comparison of the COF/AC composite with different adsorbents for cadmium and copper removal

Analyte	Adsorbent	Samples	Amount	pH	Time	Removal	Ref
Cadmium	Modified steel-making slag	Water	1 g	4	480 min	99.10%	[42]
	Steel slag	Water and wastewater	3 g	10	60 min	99%	[43]
	Sunflower waste carbon	Water and wastewater	2 g	6	180 min	99.90%	[44]
	Zeolite X	Water and wastewater	0.4 g	7.5	35 min	99.96%	[45]
	COF/AC composite	Water and wastewater	0.22 g	5	20 min	93.46%	This study
Copper	Bioballs	Water and wastewater	2 g	6.2	45 min	78%	[46]
	*Azadirachta indica* powder	Water and wastewater	1 g	7	60 min	88.90%	[47]
	Nano hydroxyapatite	Water and wastewater	2.2 g	5.5	480 min	97.68%	[48]
	Dolochar	Water and wastewater	2 g	5	15 min	99.40%	[49]
	COF/AC composite	Water and wastewater	0.22 g	5	20 min	97.45%	This study

## Conclusion

This study explored the possible COF/AC composite use with the adsorption method for cadmium and copper removal from aqueous solutions. The COF/AC composite was characterized using SEM, BET, XRD, VSM, and EDX techniques. Then, the effects of different operational parameters, including heavy metal concentration, composite content, pH, and ultrasound radiation time, were evaluated to obtain optimal conditions for the removal process. The SEM and XRD techniques showed the successful synthesis of the COF/AC composite, with a surface area of 659.4 m^2^ g^−1^, an average diameter of 3.6 nm, and a pore volume of 0.482 cm^3^ g^−1^. The results showed that all variables significantly (p < 0.05) affected the responses. Optimal conditions for removal by the COF/AC composite were obtained with the ultrasound radiation time of 20 min, pH of 5, composite content of 0.22 g, and a concentration of 19 mg L^−1^. Reusing the COF/AC composite up to 4 times did not significantly lower removal efficiency, indicating the stability and high efficiency of the adsorbent. The analysis of real samples demonstrated that cadmium and copper removal rates were 89.62% and 96.37%, respectively. Therefore, the matrix of the samples did not significantly influence the performance of the proposed method. The results of this study show that the COF/AC composite effectively removes cadmium and copper, with good potential for treating wastewater contaminated with these metals.

## Supplementary Information

Supplementary material 1

## Data Availability

All data generated or analyzed during this study are included in this published article.
