# Supplementary material for: Optimization of variables for cadmium and copper removal using magnetic nanocomposite
Source: BMC Chem. 2025 May 18;19(1):132. doi: 10.1186/s13065-025-01502-5 (PMC12087181; doi:10.1186/s13065-025-01502-5)
Supplement: Supplementary file 1 — Supplementary material 1 [file 13065_2025_1502_MOESM1_ESM.docx]

**Supplementary Material**

**Title**

**Optimization of variables for cadmium and copper removal using magnetic nanocomposite**

**Figures captions**

**Fig. S1.** a) SEM, b) EDX, c) BET, d) BJH, e) XRD, and f) VSM of COF/AC composite.

**Fig. S2.** Plot of residuals versus predicted for a) cadmium and b) copper; Normal plots for c) cadmium and d) copper; Plot of predicted versus actual for e) cadmium, and f) copper.

**Tables captions**

**Table S1.** Optimum conditions of remove heavy metals.

| 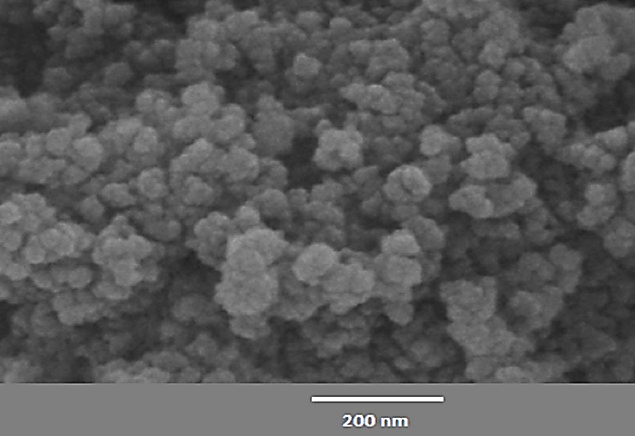  a) | 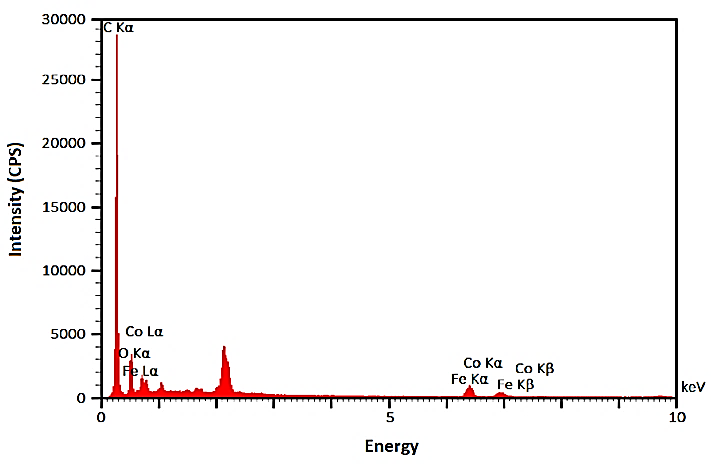  b) |
| --- | --- |
| 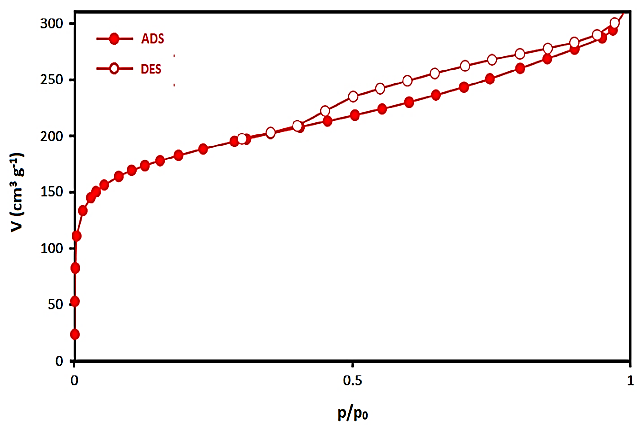  c) | 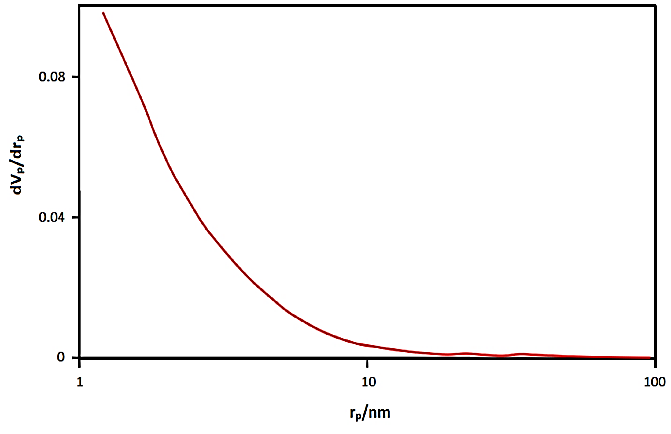  d) |
| 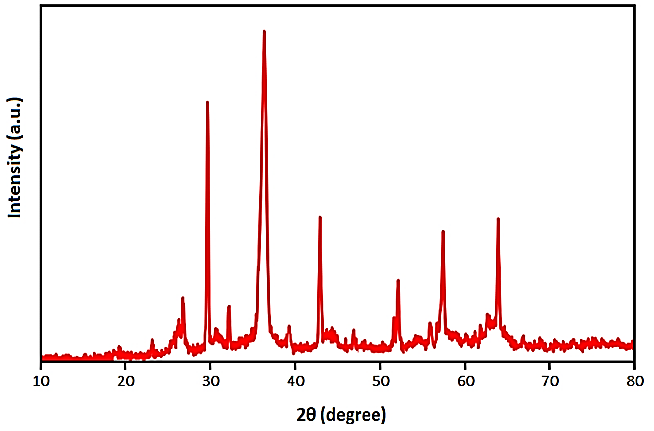  e) | 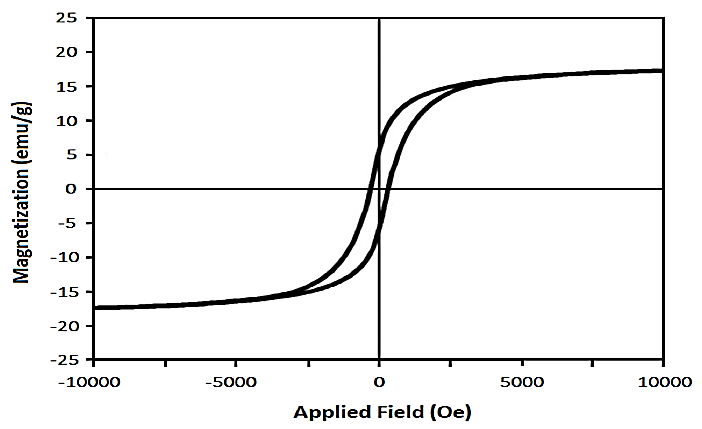  f) |
| **Fig. S1.** a) SEM, b) EDX, c) BET, d) BJH, e) XRD, and f) VSM of COF/AC composite. | |

| 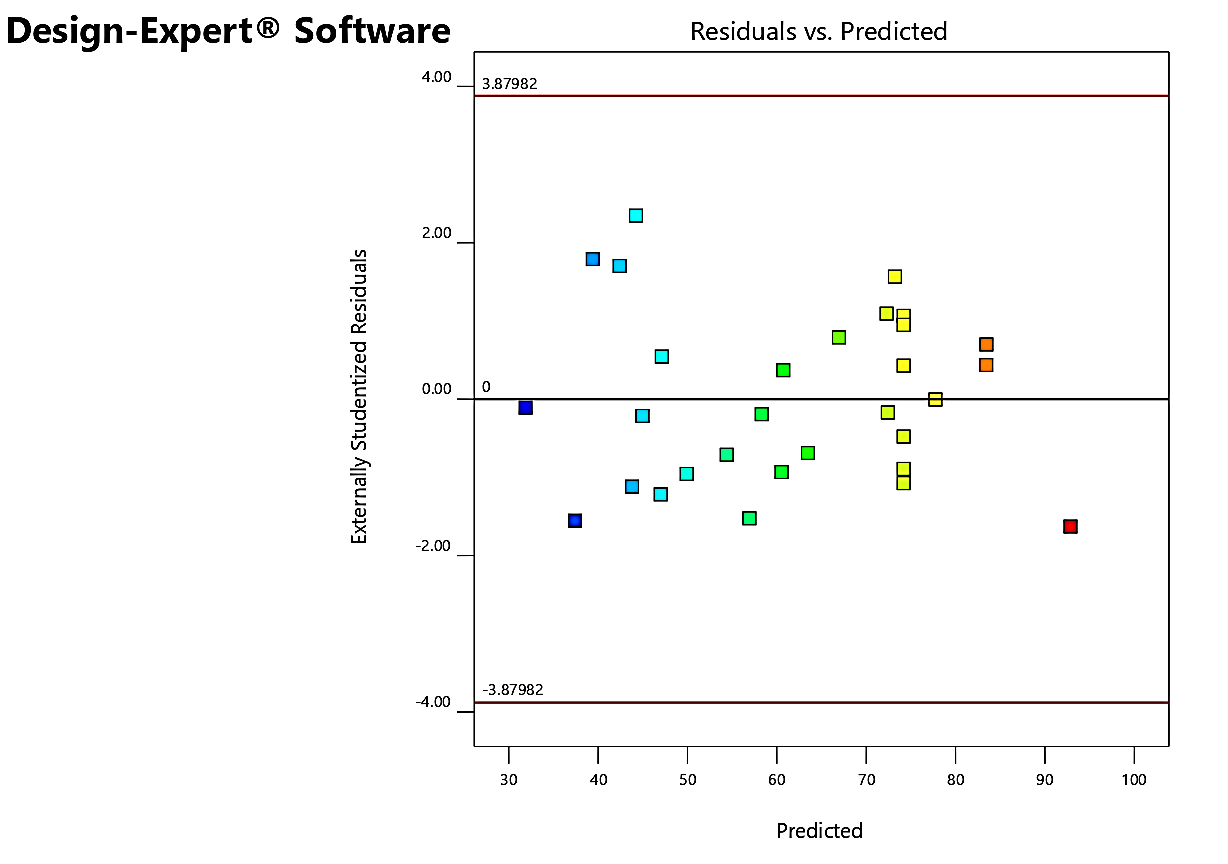  b)  a) | 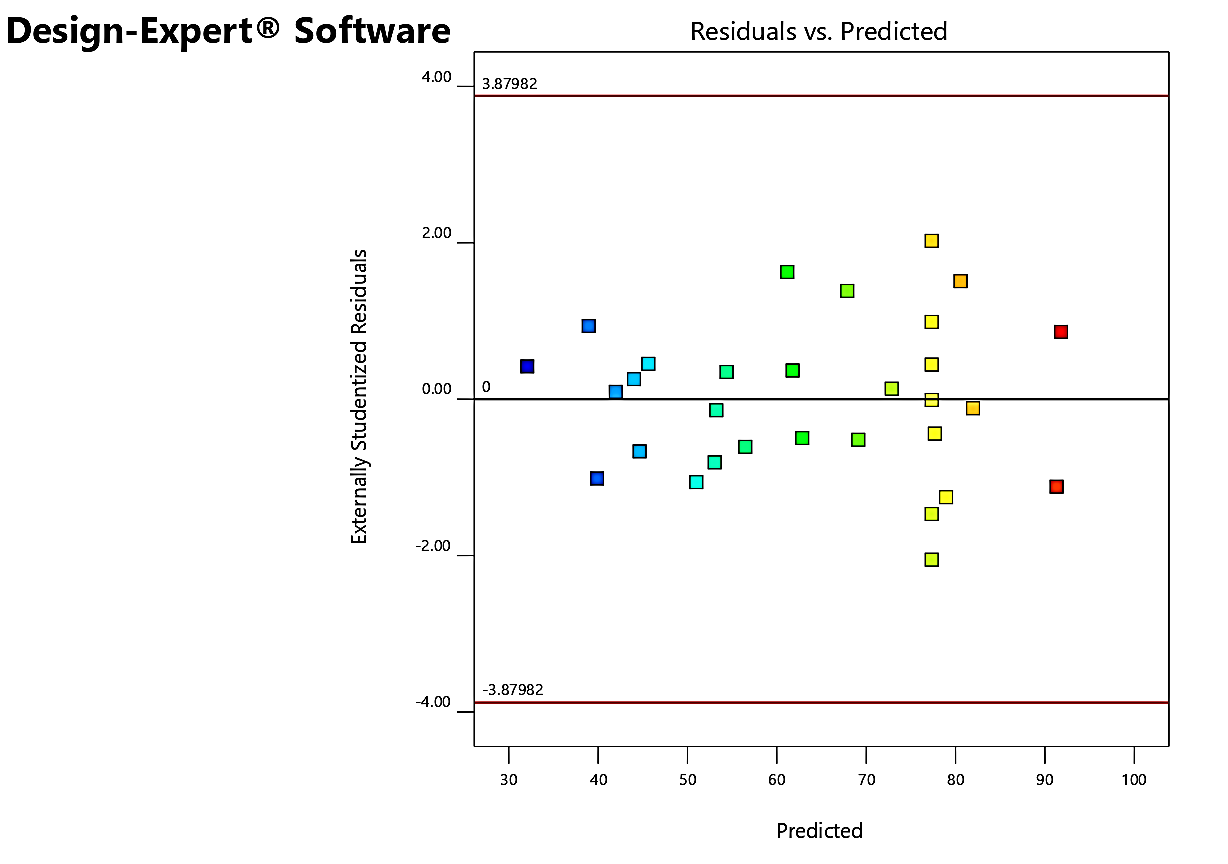 |
| --- | --- |
| 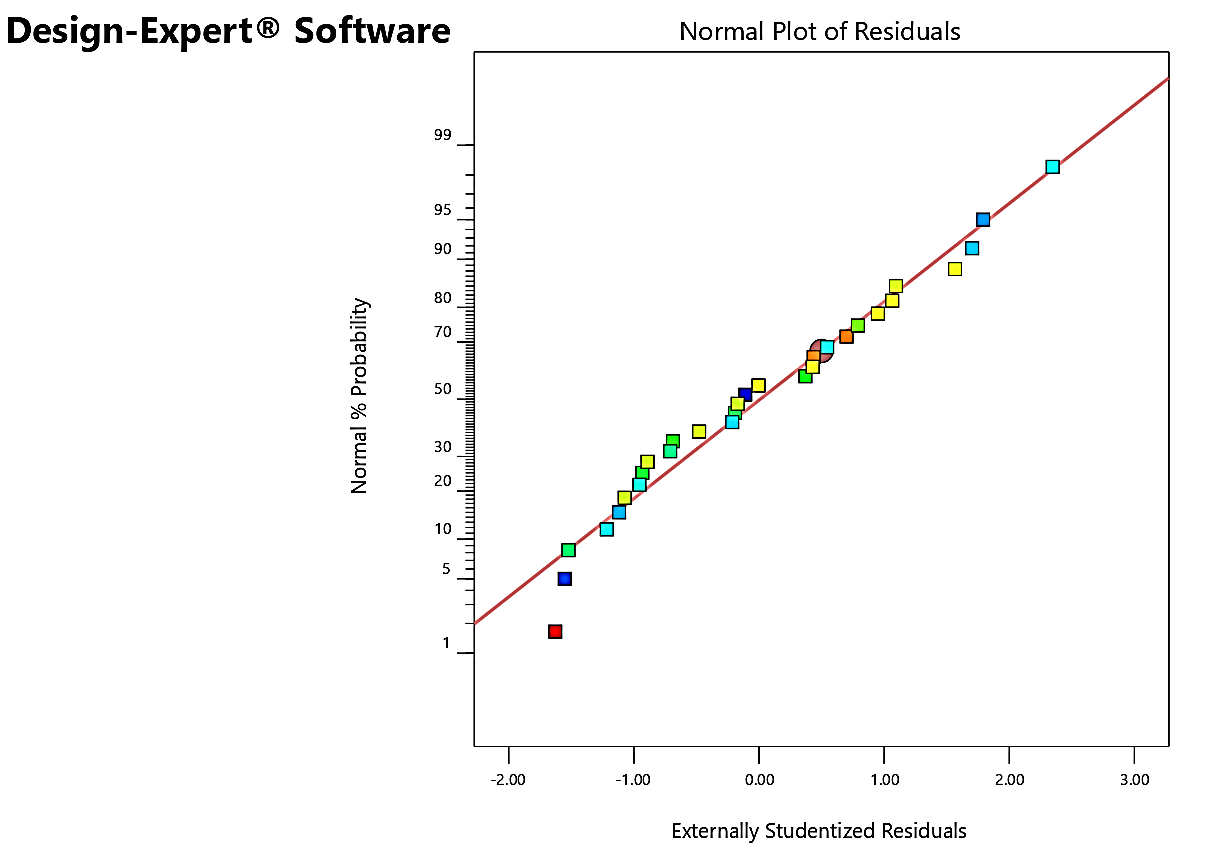  c) | 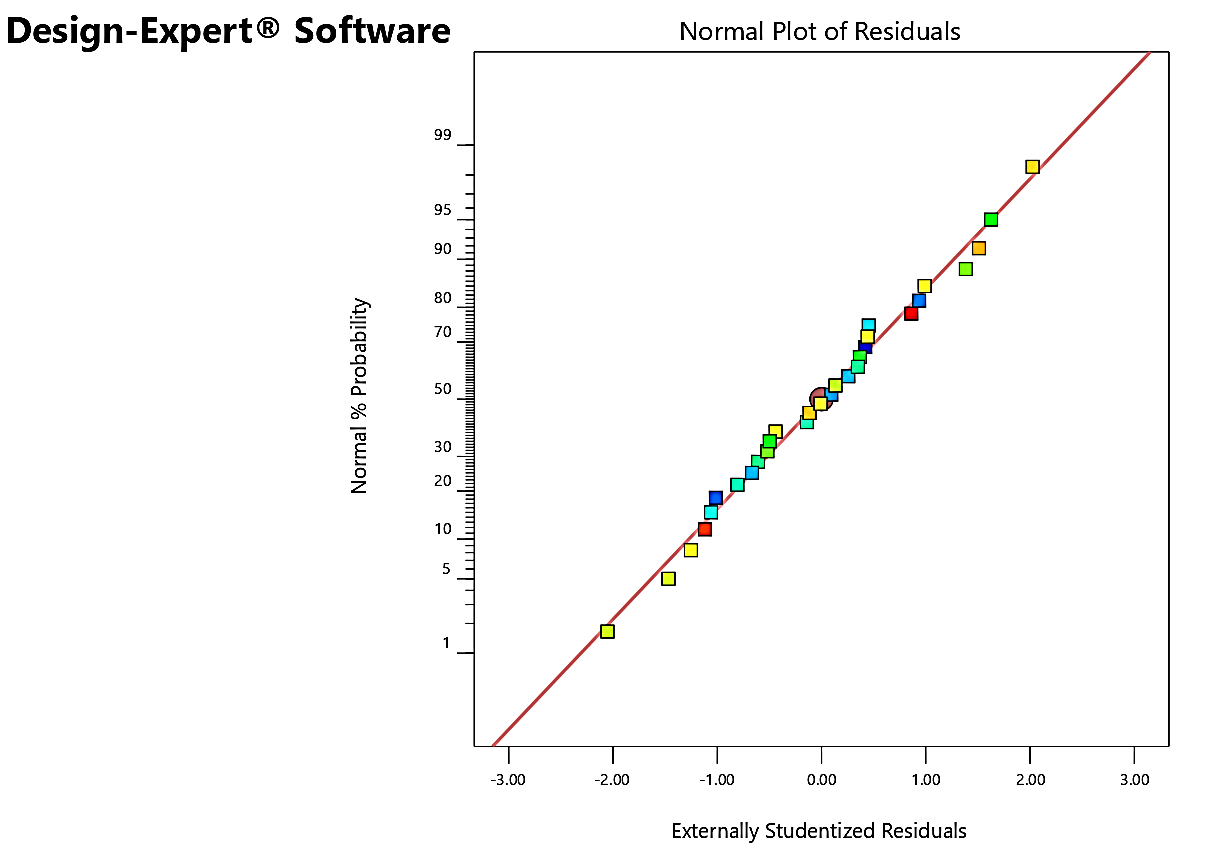  d) |
| 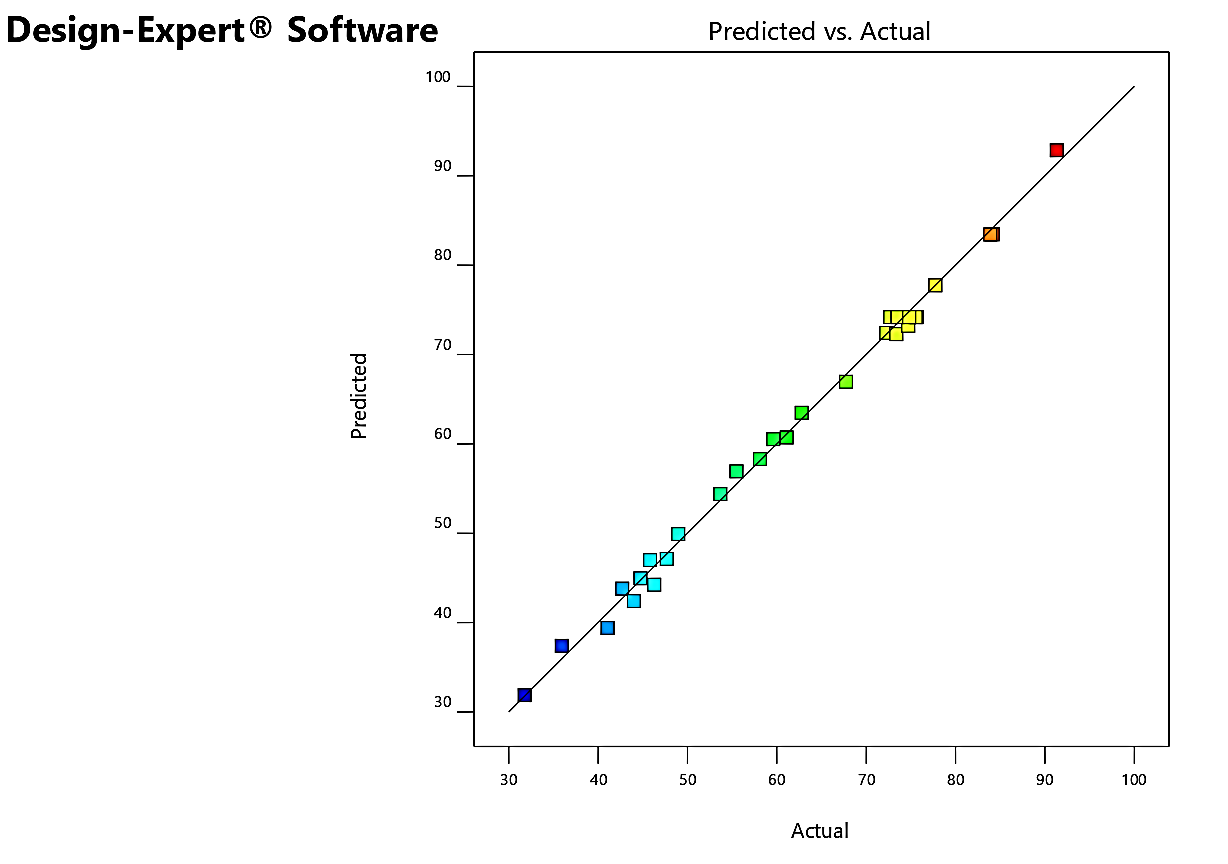  e) | 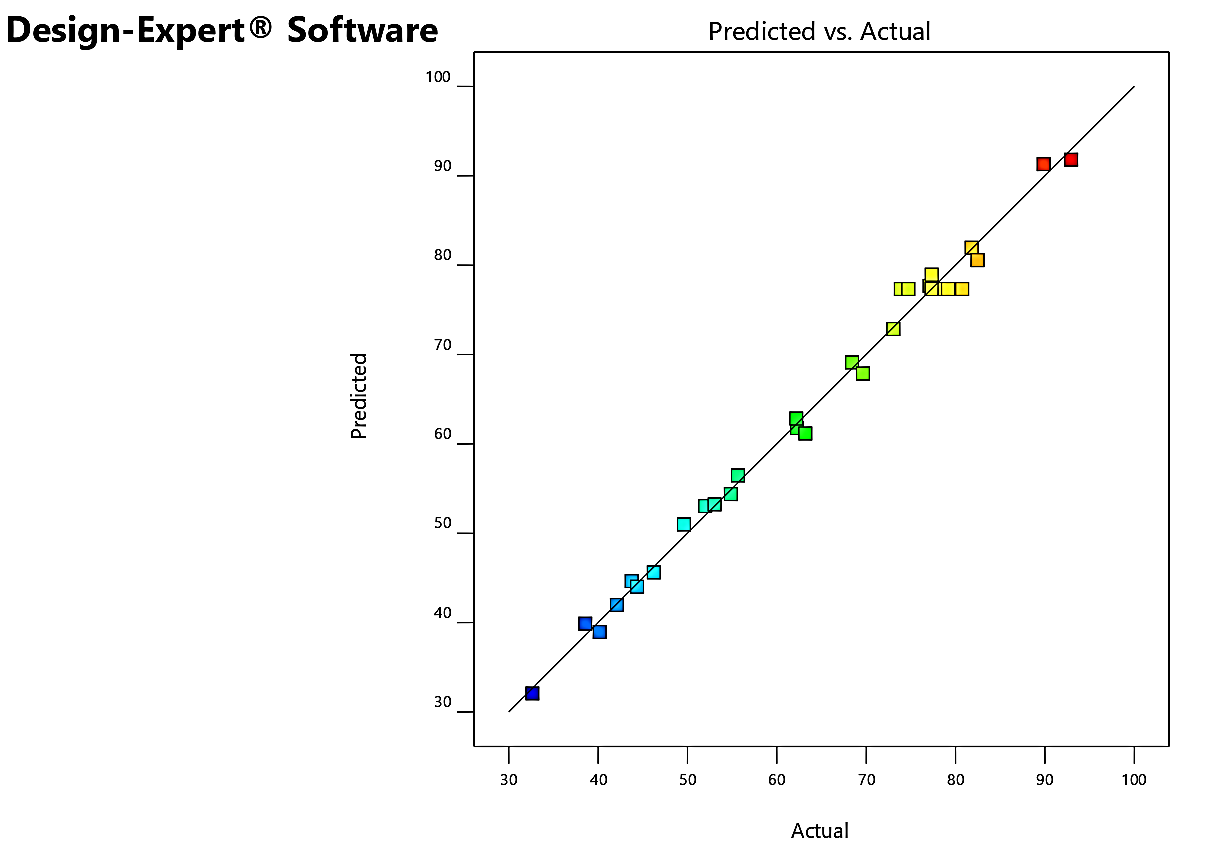  f) |
| **Fig. S2.** Plot of residuals versus predicted for a) cadmium and b) copper; Normal plots for c) cadmium and d) copper; Plot of predicted versus actual for e) cadmium, and f) copper. | |

**Table S1.** Optimum conditions of remove heavy metals.

| Optimal conditions | | | |  | | %Removal | | | |
| --- | --- | --- | --- | --- | --- | --- | --- | --- | --- |
|  |  |  |  |  | | Cadmium | | Copper | |
| Run | A (mg L^-1^) | B | C (g) | D (min) |  | Experimental | Predicted | Experimental | Predicted |
| 1 | 19 | 5 | 0.22 | 20 |  | 93.46 | 92.85 | 95.81 | 95.27 |
| 2 | 19 | 5 | 0.22 | 20 |  | 92.90 | 92.85 | 97.45 | 95.27 |
| 3 | 19 | 5 | 0.22 | 20 |  | 91.79 | 92.85 | 96.38 | 95.27 |
